# Nuclear export signal (NES) of transposases affects the transposition activity of mariner-like elements *Ppmar1* and *Ppmar2* of moso bamboo

**DOI:** 10.1186/s13100-019-0179-y

**Published:** 2019-08-19

**Authors:** Muthusamy Ramakrishnan, Ming-Bing Zhou, Chun-Fang Pan, Heikki Hänninen, Ding-Qin Tang, Kunnummal Kurungara Vinod

**Affiliations:** 1 State Key Laboratory of Subtropical Silviculture, Zhejiang A&F University, Lin’an, Hangzhou, 311300 Zhejiang Province People’s Republic of China; 2 Zhejiang Provincial Collaborative Innovation Center for Bamboo Resources and High-efficiency Utilization, Zhejiang A&F University, Lin’an, Hangzhou, 311300 Zhejiang Province People’s Republic of China; 3 Division of Genetics, Rice Breeding and Genetics Research Centre, ICAR-Indian Agricultural Research Institute, Aduthurai, Tamil Nadu 612101 India

**Keywords:** Mariner-like elements (MLEs), Nuclear export signal (NES), Transposase, Transposition activity, *Ppmar1*, *Ppmar2*, Moso bamboo

## Abstract

*Ppmar1* and *Ppmar2* are two active *mariner*-like elements (MLEs) cloned from moso bamboo (*Phyllostachys edulis* (Carrière) J. Houz) genome possessing transposases that harbour nuclear export signal (NES) domain, but not any nuclear localization signal (NLS) domain. To understand the functions of NES in transposon activity, we have conducted two experiments, fluorescence and excision frequency assays in the yeast system. For this, by site-directed mutagenesis, three NES mutants were developed from each of the MLE. In the fluorescence assay, the mutants, *NES-1*, *2* and *3* along with the wild types (*NES-0*) were fused with fluorescent proteins, enhanced yellow fluorescent protein (EYFP) and enhanced cyan fluorescent protein (ECFP) were co-transformed into yeast system. To differentiate protein localisation under the NES influence, ECFP alone was fused to wild and mutant NES domains either on N- or C-terminal and not to EYFP. Fluorescence assay revealed that blue fluorescence of ECFP was more intense than the red fluorescence of the EYFP in the yeast cell matrix. Further, ECFP had a wider localisation in the cellular matrix, but EYFP was largely located in the nucleus. The *NES-1* domain was related to the comparatively high spread of ECFP, while *NES-2* and *NES-3* indicated a low spread, implying that NES activity on nuclear export increased when the NES is made leucine-rich, while the signalling activity was reduced when the leucine content was lowered in the NES domain. In the transposon excision assay, the mutant and wild type NES of both the *Ppmar* elements were integrated into an *Ade2* vector, and within the *Ade2* gene. Co-transformation of the vector together with non-autonomous *Ppmar* transposons and NES-lacking transposases was used to assess the differential excision frequencies of the mutants NES domains. In both the MLEs, *NES-1* had the highest excision suppression, which was less than half of the excision frequency of the wild type. *NES-2* and *NES-3* elements showed, up to three times increase in transposon excision than the wild types. The results suggested that NES is an important regulator of nuclear export of transposase in *Ppmar* elements and the mutation of the NES domains can either increase or decrease the export signalling. We speculate that in moso bamboo, NESs regulates the transposition activity of MLEs to maintain the genome integrity.

**Electronic supplementary material:**

The online version of this article (10.1186/s13100-019-0179-y) contains supplementary material, which is available to authorized users.

## Background

Transposable elements (TEs), or ‘jumping genes’ or transposons, are DNA sequences that have the ability to move within the genome [1]. Transposons are ubiquitous in plant and animal genomes in abundance. There are two distinctive types, (a) DNA transposons (class II) that transpose by a DNA-mediated “cut-paste” mechanism [2] and (b) retrotransposons (class I) that act through the “copy-paste” mechanism involving an RNA intermediate [3]. There are several variants within each of the two types. One of the most prevalent DNA transposon families in eukaryotic genomes is the *Tc1/mariner* superfamily, which plays a significant role in genome evolution [4–6]. Because of its near-identical sequence similarity to the bacterial insertion sequence, *IS630* [7], *Tc1/mariner* superfamily is expanded to include *IS630* elements and is renamed as *ITm* (*IS630-Tc1-mariner*) superfamily. *ITm* transposons are characterized by self-driven mobility of its members and are generally independent of host factors to mediate transposition. In nature, they show a widespread distribution*,* frequent and total random insertions, and have a high frequency of heterologous transposition [8, 9]. Because of their versatile nature, they are used in genetic studies, as a tool in gene tagging, transgenesis and insertional mutagenesis [8, 9]. *ITm* transposons are further classified [10], among which three major families are, *Tc1-like* elements (*TLEs*) [11], *mariner-like* elements (MLEs) [12] and *Pogo-like elements* [13]. Among the MLEs, two important members, *mosaic element 1* (*Mos1*) and *Haematobia irritans mariner 1* (*Himar1*) [14] are widely studied for their cross genome transportability and used as tools in genetic studies. These hyperactive elements are found to increase their transposition activities when expressed in different host genomes such as bacteria by 200–800 [15] times, and by 10–50 times when mutated [16].

MLEs have a relatively simple structure, consisting mainly of terminal inverted repeats (TIRs) and an open reading frame (ORF) [17]. Their TIRs are generally 10–40 bp long and contain protein-binding elements. ORF is 1000–1500 bp long and encodes for the transposase (TPase) gene. TPases are enzymes responsible for transposition activity. Structurally, they contain a DNA binding domain and a catalytic domain [17]. Additionally, the TPase contains one or more short sequences of nuclear localization signals (NLS) [18] and nuclear export signals (NES) [19]. NES also is a short (8–15 residues) amino acid sequence consisting usually of four to five hydrophobic residues in a protein [20]. It is often leucine-rich [21]. Figuratively, TPase expression includes nuclear transcription and processing, mRNA export to the cytoplasm, protein synthesis, and protein folding and import back into the nucleus for mediating the transposition activity. Any change in these steps can affect the transposition frequency [22]. While NLS acts for nuclear retention of the TPase molecules, NES mediates export out of the nucleus. Therefore, depending on the cellular stage and physiological state, counterbalancing activities of NLS and NES motifs can influence the transposition activity [16, 23]. Hancock et al. [24] found that a mutation of the NES domain in the TPase of *mPing*, a deletion derivative of autonomous rice *PIF/Harbinger* transposon *Ping*, increased transposition activity in both yeast and plants. Similarly, Fattash et al. [19] identified that a mutation of a putative NES in the *Ozma* TPase dramatically increased transposition of the *Eif* and *Goblin* MITEs in yeast.

In our previous study, we reported an abundance of MLEs in various bamboo genomes belonging to 38 genera of the Bambusoideae family [25]. Consequently, two full-length *MLE* transposons, named *Ppmar1* and *Ppmar2,* were cloned from moso bamboo (*Phyllostachys edulis* (Carrière) J. Houz) genome [26]. Both of these transposons were demonstrated to transpose in yeast and *Arabidopsis thaliana* genome and exhibited their affinity towards TA-rich regions. They were also shown to get integrated into nearby genes [27–29]. TPases of both, *Ppmar1* and *Ppmar2* harbour NES domains, but it is still uncertain whether and how the NESs affects their transposition. In order to examine this, we used site-directed mutagenesis to mutate NES sequences of *Ppmar1* and *Ppmar2* in moso bamboo. The influence of the mutated NES domains on the localization of TPase in the cell and their transposition frequencies were systematically studied using different NES mutants by the help of a yeast screening system and yeast transposition assay. To the best of our knowledge, no earlier reports are available on the influence of NESs on TPase localization and transposition activity of *Ppmar1* and *Ppmar2* transposons.

## Methods

### Prediction of NES sequences in *Ppmar1* and *Ppmar2* transposase

The NES sequences of *Ppmar1* and *Ppmar2* TPase were predicted using NetNES 1.1 online software (http://www.cbs.dtu.dk/services/NetNES/) [30] and were named *Ppmar1-NES* and *Ppmar2-NES*, respectively. The nucleotide sequences of *Ppmar1* and *Ppmar2* TPases and their amino acid sequences are given in Additional file 1.

### Construction of NES domain with the ECFP fragments in the plasmid pPS1890

In our earlier studies, full-length sequences of *Ppmar1* and *Ppmar2* were isolated from moso bamboo leaves and were cloned into pMD18-T vector [26–28]. In this study, we used these clones for amplifying *Ppmar1* and *Ppmar2*. Using an overlap PCR, the NES sequences of the TPases were fused to the C-terminal and N-terminal of the enhanced cyan fluorescent protein (ECFP) (730 bp) of the pPS1890 plasmid DNA that also contained an NLS domain [31]. The constructed plasmids were named pPS1890-NES-ECFP (NES fused in the N-terminal of ECFP) and pPS1890-ECFP-NES (NES fused in the C-terminal of ECFP). The primer sequences used to amplify the ECFP-NLS are given in Additional file 2.

### Mutations in NES domains of *Ppmar1* and *Ppmar2* transposases

Based on the NES domain sequences of *Ppmar1* and *Ppmar2*, three types of NES domains were designed using NetNES 1.1 for mutating their amino acid sequences. This was done in order to obtain a strong NES signal (NES-1), a weak NES signal (NES-2), and an intermediate NES signal (NES-3) (Table 1). By the use of QuikChange Lightning Site-Directed Mutagenesis Kits (Stratagene, USA) TPase NES sequences in the plasmids pPS1890-NES-ECFP and pPS1890-ECFP-NES were mutated to generate the three types of NES domains. All mutated sequences were sequenced for confirmation. The primer sequences used to mutate the NES sequences of TPases are given in Additional file 2. The mutated domains were designated as *Ppmar1-NES-1, Ppmar1-NES-2*, *Ppmar1-NES-3, Ppmar2-NES-1*, *Ppmar2-NES-2* and *Ppmar2-NES-3*. The wild types were correspondingly designated as *Ppmar1-NES-0* and *Ppmar2-NES-0*.

**Table 1:** Amino acid mutations in NES domains of *Ppmar1-NES* and *Ppmar2-NES* transposases of moso bamboo

Names of NES	NES type	Sequence	Mutated site	Approximate NES score
*Ppmar1-NES-0*	Wild type NES	KQRLEREDRLPLQIP	–	0.9
*Ppmar1-NES-1*	Strong NES	K**L**RLEREDRLPLQIP	Q → L	1
*Ppmar1-NES-2*	Weak NES	KQRLEREDR**A**P**A**QIP	L → A; L → A	0
*Ppmar1-NES-3*	Intermediate NES	KQRLEREDR**AEI**QIP	L → A; P → E; L → I	0.5
*Ppmar2-NES-0*	Wild type NES	RNGVLSIRLQCDL	–	0.85
*Ppmar2-NES-1*	Strong NES	R**L**GVLSIRLQCDL	N → L	1
*Ppmar2-NES-2*	Weak NES	RNGVLS**A**RLQCDL	I → A	0
*Ppmar2-NES-3*	Intermediate NES	RNGVL**L**IRLQCDL	S → L	0.5

The bold red letters stand for mutated amino acids

Letters on the left- and right-hand sides of the arrows indicate the wild type and the mutated amino acid, respectively. The NES types were identified based on the NES scores

*Q* glutamine, *L* leucine, *A* alanine, *P* proline, *E* glutamic acid, *N* asparagine, *I* isoleucine, *S* serine

### Yeast ECFP fluorescence screening

Two vectors, pPS1888 and pPS1890 (Addgene, Cambridge, USA), were used for yeast transformation. The pPS1888 contained an enhanced yellow fluorescent protein (EYFP), while the vector pPS1890 had ECFP. On transformation, yeast cells carrying pPS1888 emits a yellow-green fluorescence (527 nm) under 513 nm excitation, while the cells carrying the pPS1890 emits blue fluorescence (475 nm) under 433 nm excitation, both in the cell nucleus. Both of the vectors carry an NLS domain upstream of the fluorescent protein. In order to distinguish yellow-green fluorescence from blue fluorescence, yellow-green fluorescence was converted into red fluorescence using a Zeiss LSM 510 META laser scanning confocal microscope (Zeiss, Germany) [32].

The pPS1890 vector was recombined both with wild-type and mutant NES sequences. The recombined pPS1890 vector and the unrecombined pPS1888 vector were transformed into yeast. After incubating at 30 °C for 10 days, a single colony was selected for fluorescence observations by a Zeiss LSM 510 META laser scanning confocal microscope. The individual channels of ECFP (blue) and EYFP (red) were merged to produce the final images using Zeiss LSM Software ZEN 2009 of a confocal microscope. The intensity of both blue and red fluorescence was quantified in each channel of ECFP and EYFP, respectively using ImageJ v.1.5.2a [33]. The red fluorescence emitted by the pPS1888 vector is known to occur in the nucleus, so based on the relative localization of blue and red fluorescence, the distribution of the modified ECFP in cells was determined.

### Construction of pAG413gal-Tpase1 and pAG413gal-Tpase2 vectors having NES sequences

TPase sequences of *Ppmar1* and *Ppmar2* containing NES sequences were amplified with *Not* I and *EcoR* V sites added. The amplification fragments were digested by *Not* I and *EcoR* V enzymes. The pAG413gal-ccdB vector with *His3* selectable marker was also cut by both restriction enzymes, and the big fragment was recovered. Then the digested TPase fragments and the backbone of pAG413gal-ccdB were ligated by T_4_ DNA ligase, resulting in the recombined vectors pAG413gal-Tpase1 and pAG413gal-Tpase2. The TPase was promoted to be expressed under the *gal* promoter. Using QuikChange Lightning Site-Directed Mutagenesis Kit the NES sequences in the TPase sequences of pAG413gal-Tpase1 and pAG413gal-Tpase2 were mutated into three versions corresponding to the three NES types (Table 1). The primer sequences are provided in Additional file 2.

### Construction of non-autonomous pWL89A-*Ppmar1NA* and pWL89A-*Ppmar2NA* vectors

The pMD18-T vector [28] containing full-length *Ppmar1,* including two target site duplicates (TSDs) of dinucleotide Thymine (T) and Adenine (A), was cut by *BseR* I and the resultant 5′ and 3′ TIRs of *Ppmar1* and their adjacent sequences were ligated together leading to truncated *Ppmar1NA*. The *Ppmar1NA* was 778 bp long consisting of the TIRs, TSDs and the sub-terminal sequences without TPase. The sequence of *Ppmar1NA* is indicated in Additional file 3. Simultaneously, the non-autonomous transposon of *Ppmar2, Ppmar2NA,* was also constructed (Additional file 3). The 5′ and 3′ TIRs and their adjacent sequences of *Ppmar2* were amplified. The details of the primers (Mini-s-1-20-1F and Mini-s-1-20-1R1) used for amplifying the 5’TIR region and the primers (Mini-s-1-20-2F1 and Mini-s-1-20-2R) used for amplifying the 3’TIRs region are given in Additional file 4. Combining the resultant fragments amplified by the upstream primer (Mini-s-1-20-1F) and the downstream primer (Mini-s-1-20-2R), resulted in the *Ppmar2NA.* The sequence of *Ppmar2NA* is indicated in Additional file 3.

*Ppmar1NA* and *Ppmar2NA* were inserted into the *Xho* I site at the 5′ untranslated region (UTR) of the *Ade2* gene in the vector pWL89A possessing two selectable markers of *Ura3* and *Ade2*. This resulted in two recombined vectors, named pWL89A-*Ppmar1NA* and pWL89A-*Ppmar2NA*, respectively.

### Yeast transposition assay of excision frequencies

In order to quantify the transposon excision frequency (TEF) catalyzed by mutated TPases in the yeast cells, six separate yeast transposition assays were performed. Both of the pairs of vectors (pAG413gal-Tpase1 and pWL89A-*Ppmar1NA*; pAG413gal-Tpase2 and pWL89A-*Ppmar2NA*) were transformed into yeast strain DG2523 (MAT-alpha Ura3–167 Trp1-HisG Leu2-HisG His3-Del200 Ade2-HisG). After double transformation, yeast strains were grown on a complete supplement mixture (CSM) medium lacking histidine and uracil (CSM-his-ura) with 2% galactose at 30 °C for 10 days. Single colonies were then dissolved in 150 μl water and plated on a medium lacking adenine, histidine and uracil (CSM-ade-his-ura) and having 2% galactose as the sole carbon source. The plates were incubated at 30 °C for 20 days to allow the growth of revertant colonies.

In the yeast assay *Ade2*, revertant frequencies were counted for the wild type and the NES mutated TPase constructs. In detail, the cells in each galactose-induced colony were suspended in 50 μl of water and plated on a media lacking adenine. Growth of the yeast cells on medium lacking adenine required the excision of transposon and the expression of the *Ade2* gene. An equal volume, but diluted 1 × 10^5^ times from the cell suspension mentioned above, was placed on CSM-ade-his-ura medium to obtain the total number of viable cells in the galactose-induced colony. Six separate experiments were carried out and the excision frequencies obtained from them were averaged in the analysis of the results.

## Results

### Prediction of NES of *Ppmar1* and *Ppmar2* transposases

The NES sequences of TPases were predicted to be located at the positions at 471–481 bp (score = 0.90) for *Ppmar1* and 435–447 bp for *Ppmar2* (score = 0.85). They had sequence lengths of 120 bp and 126 bp respectively with amino acid sequences of KQRLEREDRLPLQIP and RNGVLSIRLQCDL. Both the NES domains were leucine-rich with constituent hydrophobic amino acids such as isoleucine (I), proline (P), glycine (G), valine (V) and leucine (L). Evaluated by the software NetNES 1.1, three types of NES domains were predicted for mutation with strong, intermediate and weak transport signals and having predicted scores close to 1.0, 0.5 and 0.0, respectively. Details of amino acid mutations and scores are given in Table 1. The NES scores close to 1.0 (*Ppmar1-NES-1* and *Ppmar2-NES-1*) are considered to have strong regulation of TPase export from the nucleus. The intermediate NES scores close to 0.5 (*Ppmar1-NES-3* and *Ppmar2-NES-3*) are considered to have a moderate level of ex-nuclear export while the signal scores close to 0.0 (*Ppmar1-NES-2* and *Ppmar2-NES-2*) are considered to have a weak regulation.

### Differential fluorescence in mutant yeast cells

**Fig. 1:**
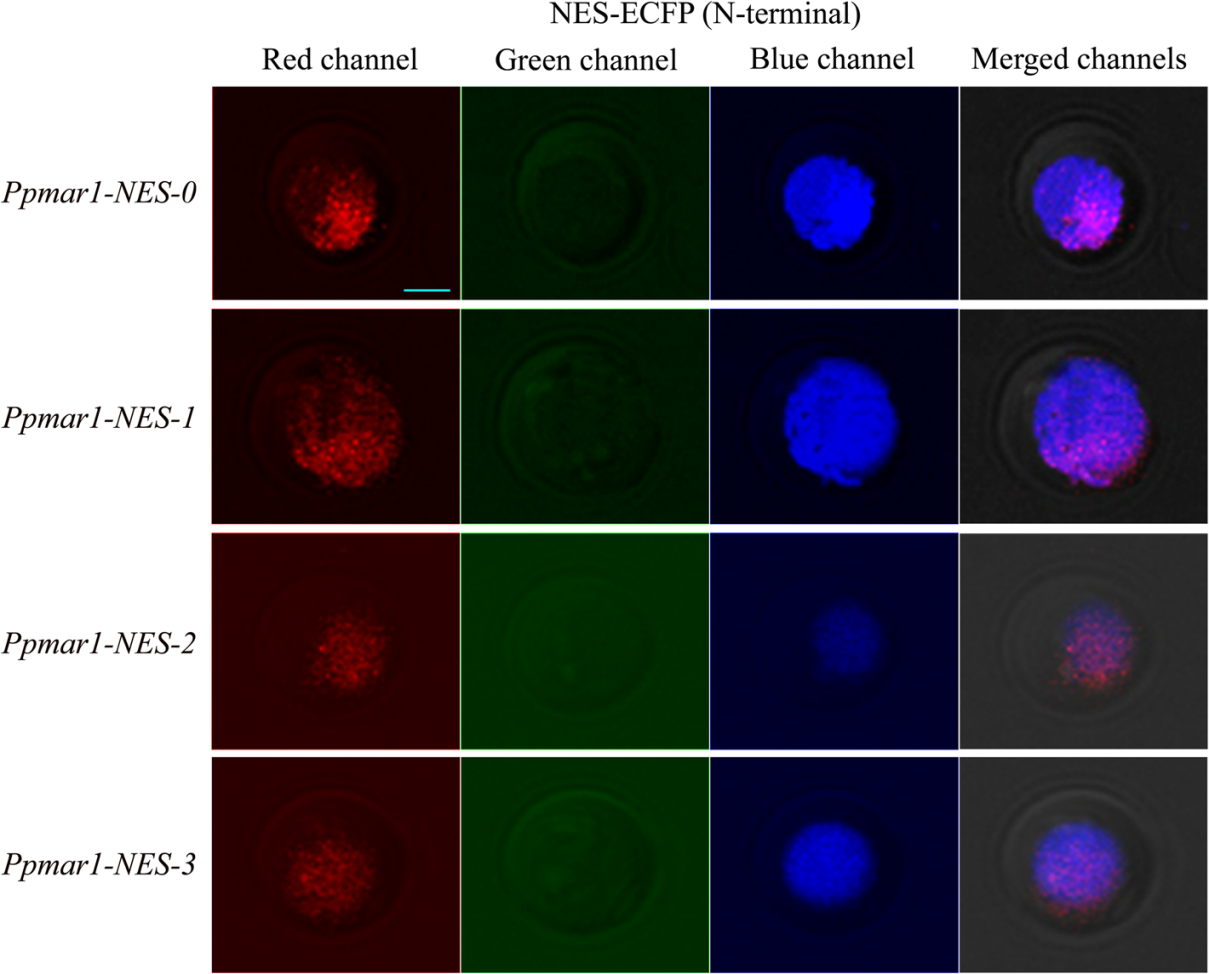
The localization signal of reporter proteins fused to various *Ppmar1-NES* transposase in yeast. Fluorescence observation of ECFP fused with NES of *Ppmar1* transposase in N-terminal (NES-ECFP). The photographs illustrate the microscopy of the red channel, green channel and blue channel and merged channels of yeast expressing ECFP fused into different versions of NES. The nuclear translocation EYFP (yellow-green) was co-expressed to provide a nuclear marker. In order to differentiate yellow-green fluorescence from blue fluorescence, yellow-green fluorescence was converted into red fluorescence. Scale bar indicates 5 μm

**Fig. 2:**
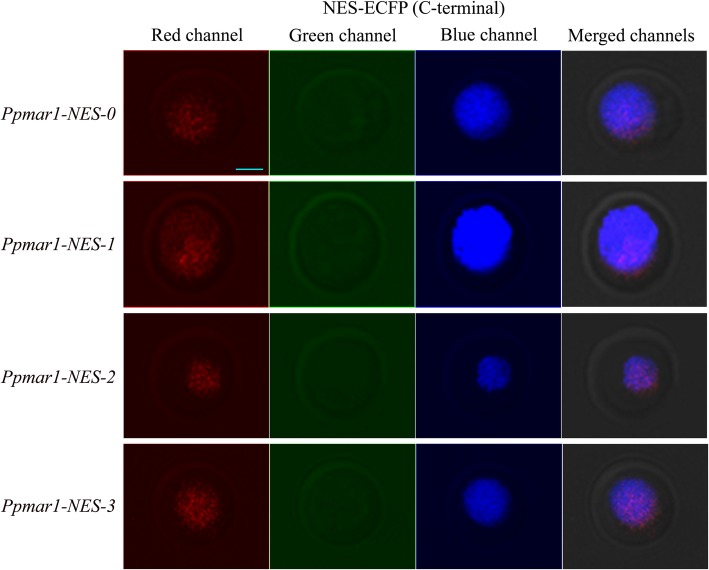
The localization signal of reporter proteins fused to various *Ppmar1-NES* transposase in yeast. Fluorescence observation of ECFP fused with NES of *Ppmar1* transposase in C-terminal (ECFP-NES). The photographs illustrate the microscopy of the red channel, green channel and blue channel and merged channels of yeast expressing ECFP fused into different versions of NES. The nuclear translocation EYFP (yellow-green) was co-expressed to provide a nuclear marker. In order to differentiate yellow-green fluorescence from blue fluorescence, yellow-green fluorescence was converted into red fluorescence. Scale bar indicates 5 μm

**Fig. 3:**
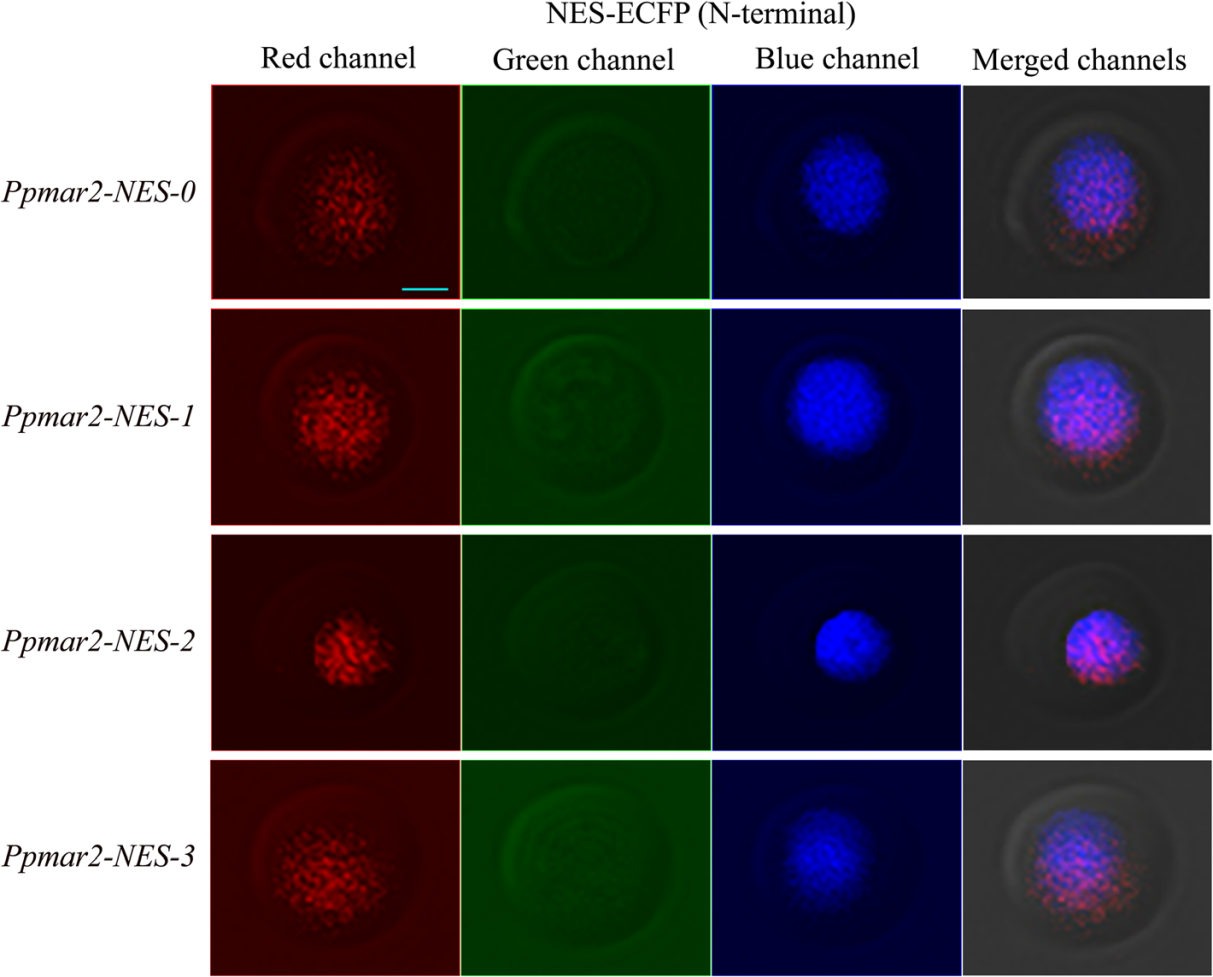
The localization signal of reporter proteins fused to various *Ppmar2-NES* transposase in yeast. Fluorescence observation of ECFP fused with NES of *Ppmar2* transposase in N-terminal (NES-ECFP). The photographs illustrate the microscopy of the red channel, green channel and blue channel and merged channels of yeast expressing ECFP fused into different versions of NES. The nuclear translocation EYFP (yellow-green) was co-expressed to provide a nuclear marker. In order to differentiate yellow-green fluorescence from blue fluorescence, yellow-green fluorescence was converted into red fluorescence. Scale bar indicates 5 μm

**Fig. 4:**
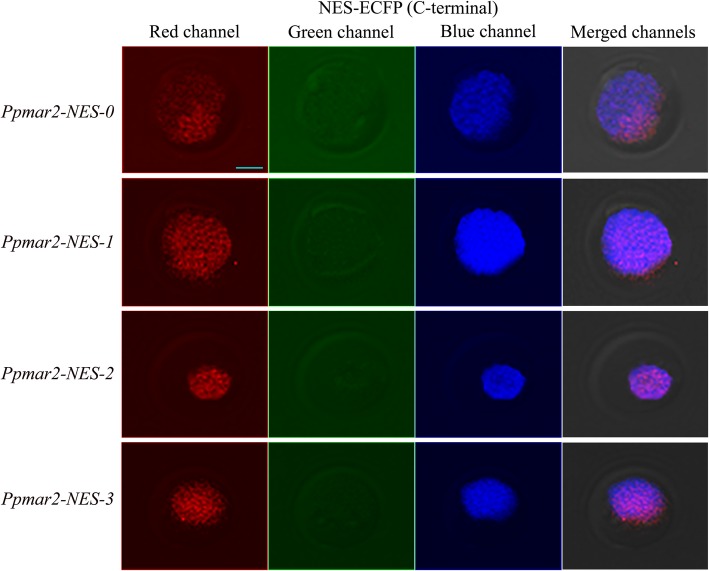
The localization signal of reporter proteins fused to various *Ppmar2-NES* transposase in yeast. Fluorescence observation of ECFP fused with NES of *Ppmar2* transposase in C-terminal (ECFP-NES). The photographs illustrate the microscopy of the red channel, green channel and blue channel and merged channels of yeast expressing ECFP fused into different versions of NES. The nuclear translocation EYFP (yellow-green) was co-expressed to provide a nuclear marker. In order to differentiate yellow-green fluorescence from blue fluorescence, yellow-green fluorescence was converted into red fluorescence. Scale bar indicates 5 μm

In all the cases, irrespective of the NES used, the yellow fluorescence emitted by the EYFP as detected by the red colour in laser scanning confocal microscopy was limited to the nucleus alone. The localization of different versions of NES fused to the ECFPs in the yeast cells were detected in the fluorescence screening system. In both of the wild-types, *Ppmar1-NES-0* and *Ppmar2-NES-0,* blue fluorescence was observed both in the nucleus and the cytoplasm, with the former displaying relatively stronger fluorescence than the latter. However, in the mutant types with weaker export signals, *Ppmar1-NES-2* and *Ppmar2-NES-2*, the blue fluorescence was only observed in the nucleus and not in the cytoplasm (Figs. 1, 2, 3 and 4). Whereas, in *Ppmar1-NES-1* and *Ppmar2-NES-1*, the mutants with stronger NES, a deeper blue fluorescence was observed in the cytoplasm than in the nucleus. In the intermediate mutant types, *Ppmar1-NES-3* and *Ppmar2-NES-3*, the blue fluorescence was rather uniformly distributed in the nucleus and the cytoplasm (Figs. 1, 2, 3 and 4).

### Quantification of fluorescence intensity

**Fig. 5:**
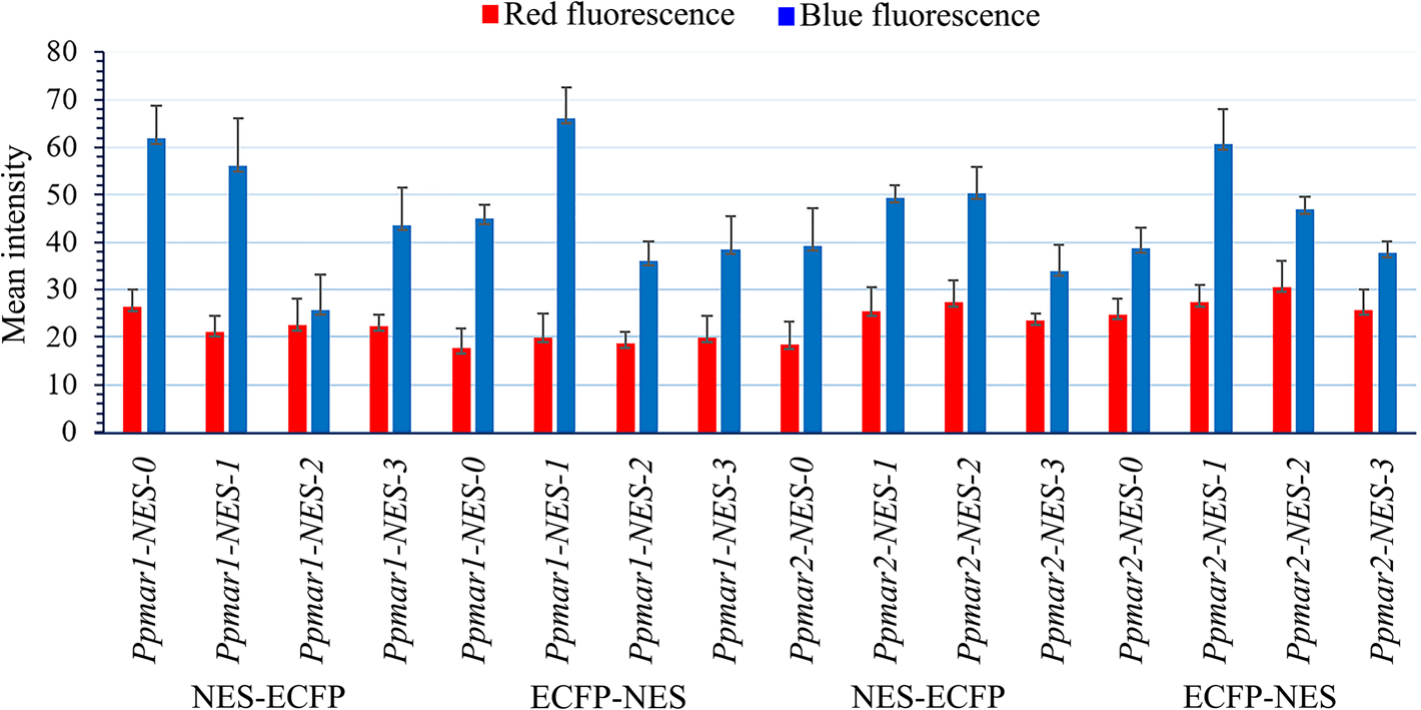
The mean intensity of fluorescence of reporter proteins fused to various *Ppmar1-NES* and *Ppmar2-NES* transposase in yeast. Blue fluorescence is ECFP fused with various NES of *Ppmar1* and *Ppmar2* transposase in N-terminal (NES-ECFP) and C-terminal (ECFP-NES). Red fluorescence is EYFP (red) was co-expressed to provide a nuclear marker

The mean pixel intensity of blue fluorescence (45.6) of ECFP fused with various *Ppmar1-NES* and *Ppmar2-NES* TPases was almost two times higher than red fluorescence (23.2) emitted by the nuclear marker protein EYFP (Fig. 5). The fluorescent intensity showed no apparent difference with the position of NES attachment to the fluorescence proteins. The expression of red fluorescence was more or less consistent (range of 17.6 to 30.5) in all *Ppmar1-NES* and *Ppmar2-NES* TPases including wild and mutants, whereas the expression of blue fluorescence varied widely (range of 25.6 to 66.0). Among the wild types, *Ppmar1-NES-0* showed a higher intensity of blue than *Ppmar2-NES-0*. In both the high-affinity mutants, *Ppmar1-NES-1* and *Ppmar2-NES-1*, the mean blue fluorescence intensity was higher than other mutants. However, in the low-affinity mutants, *Ppmar1-NES-2* and *Ppmar2-NES-2*, the pixel intensity was lower than the intermediate affinity mutants, *Ppmar1-NES-3* and *Ppmar2-NES-*3, but only when the NES was attached to the N-terminal end. When attached to the C-terminal end of the fluorescent proteins, *Ppmar1-NES-3* showed lower intensity than *Ppmar1-NES-2* (Additional file 5).

### The influence of mutated NES on the transposon excision frequency

**Fig. 6:**
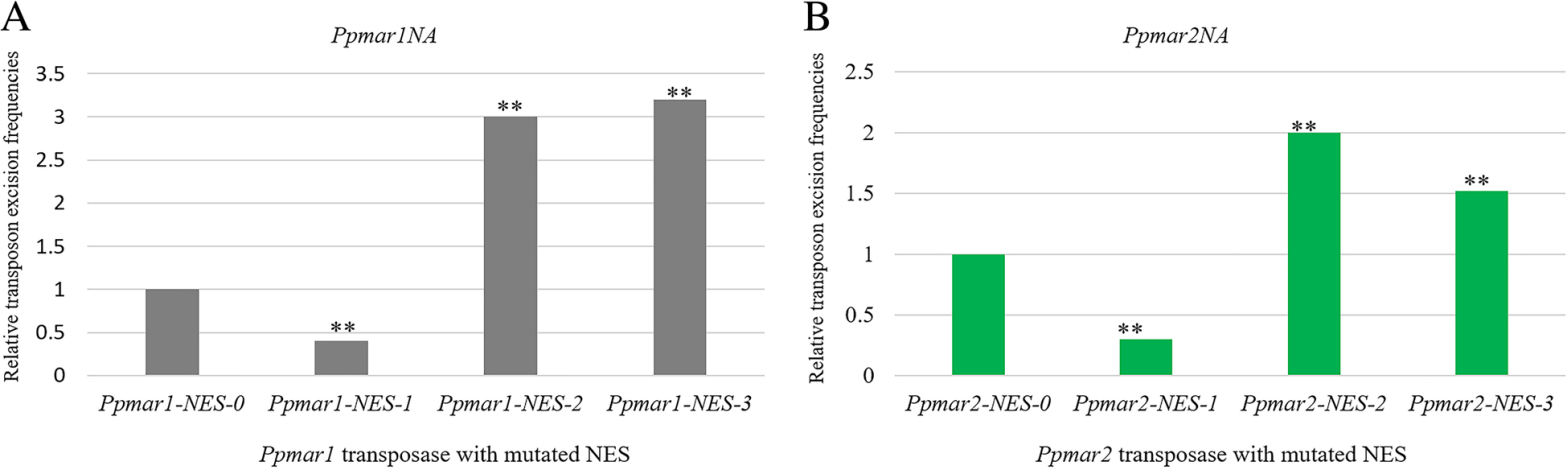
**a** Transposition frequencies of *Ppmar1NA* catalyzed by *Ppmar1* transposase with mutated NES. **b** Transposition frequencies of *Ppmar2NA* catalyzed by *Ppmar2* transposase with mutated NES. The vertical axis indicates the ratio of the mean of excision frequency catalyzed by *Ppmar1* and *Ppmar2* transposase mutants in six independent experiments, to the excision frequency catalyzed by the wild-type *Ppmar1* and *Ppmar2* transposase. Statistical significance of the differences: ** very significant (*P* < 0.01)

The TEFs by both *Ppmar1* and *Ppmar2* varied conspicuously between wild-type and the mutants that carried variation only in the NES domain (Fig. 6a and b). The non-autonomous mutants (*Ppmar1NA* and *Ppmar2NA*) catalyzed by the *NES-2* mutants of both *Ppmar1* and *Ppmar2* showed 300 and 200% higher excision frequency respectively than by the wild-types, *Ppmar1-NES-0* and *Ppmar2-NES-0*. Interestingly, the TEFs of *Ppmar1NA* and *Ppmar2NA* catalyzed by *NES-1* mutants were respectively about 45 and 40% of that the wild-type TPases. The *NES-3* mutants, however, showed 320 and 150% of the TEFs for *Ppmar1NA* and *Ppmar2NA*, in that order (Fig. 6a and b).

## Discussion

The moso bamboo MLEs, *Ppmar1* and *Ppmar2*, are active DNA transposons. The TPase enzymes of these elements show significant sequence analogy indicating their active functionality. The interesting fact that the TPase contains an NES domain and no NLS domain, signifies a potential biological role for these transposons. Although NESs have been identified in eukaryotic TPase [24], their biological functions are not yet known. However, it is well known that NESs acts in the import of transposases into the cytoplasm, thereby suppressing their excision activity. In the current study, we for the first time attempted to examine the effect of NES domain in the excision activity of *Ppmar1 and Ppmar2* and their potential role in controlling the transposition in the event of site-specific mutations.

The NESs are assumed to have an unusually significant role in moso bamboo transposons because of the absence of an NLS domain, which acts as a counterbalancing system. NLS acts in importing the TPase after synthesis from the cytoplasm to the nucleus, to incite transposition [34], while the NES facilitates export back to the cytoplasm. Therefore, a clear balance of NLS and NES activities is essential for transposon activity. Accordingly, in moso bamboo, we can assume that transposition could be largely dependent on the NES function. Since the NES actively returns the TPases back to the cytoplasm, the TPase residence time in the nucleus is reduced, thereby suppression of transposition is achieved in the resident genome.

The amino acid sequences in the NES domains are believed to play a potential role in export signalling. Our TPase mutant constructs having different export potentials in their NES domains could suggest a role for certain amino acids, such as leucine, in nuclear export signalling. Further, we have studied the activity in a yeast system for two main reasons, the first being the absence of a DNA transposon in yeast, a unique feature among all eukaryotic organisms [35] and the second being the small size of the yeast genome and its well-known genetics [36]. Additionally, the *Ppmar* transposons were successfully activated in a yeast system in earlier studies [28].

### Differential cellular localization of NES domains

In the yeast fluorescence screening system used in the present study to display the cellular localization of the NESs, the fusion to fluorescent proteins could be efficiently located using confocal microscopy. The choice of fluorescent proteins as the marker for subcellular localization owes to their easy detection in the living cells. Two fluorescence signal vectors were used, pPS1888 and pPS1890, which harboured EYFP and ECFP, respectively [37]. Since there was an NLS domain upstream from both of the fluorescent proteins in the vector, there could be an enhanced tendency of exclusive localization of both of the proteins in the nucleus. In order to differentiate their localization tendencies regulated exclusively by the presence of NLS domains, we fused NES domains only to ECFP and not to EYFP. Therefore, in the yeast system after a co-transformation of both of the vectors, we expected EYFP to remain in the nucleus, but ECFP can be either in the nucleus or in the cytoplasm, or in both because the NES domains could get ECFP exported to the cytoplasm. The localization of the fluorescent proteins was judged by the colour emitted by them in the yeast cells. The red fluorescence indicated the presence of EYFP and blue that of ECFP, and the intensity of fluorescence could be attributed to the density of the protein. When mutated NESs have differential nuclear export potential, it could affect the distribution of ECFP between nucleus and cytoplasm.

In our assays, we could see less EYFP fluorescence (red colour) than ECFP fluorescence (blue colour) in the yeast cells. The EYPF were mostly concentrated towards the nuclear region irrespective of the variation in the NES domains in the co-transformed vector, pPS1890. We assumed that preferential localisation EYFP in the nucleus could be driven by the NLS domain, since EYPF was not fused to NES, unlike that of ECFP. Additionally, the intensity of the red fluorescence remained within a narrow range in all the co-transformed systems including wild and mutants, and irrespective of the fact that the NES was attached to N- or C-terminal end of the ECFP protein in the co-transformed vector. This confirmed that the presence of EYPF was not under the influence of NES domains used in the experimental systems.

Further ascertaining the role of NES domains in the distribution of fluorescent protein, we could observe variation in the presence of blue fluorescence from ECFP in both the nucleus and the cytoplasm, although distinct quantification between these could not be done. When the wild type NES was fused to the protein, there was a high intensity of blue colour across the cell but the red colour was located only at the nuclear regions in all the four systems, that involved two different *Ppmar* elements (*Ppmar1* and *Ppmar2*) and two different fusion systems (N-terminal and C-terminal). This suggested that the fluorescent proteins fused to NES domains had higher mobility across the cell matrix, which could be due to their active export from the nucleus to the cytoplasm. Further, a relatively wider spread of the blue fluorescence across the yeast cell suggested that the influence of the NES domain on the export process was relatively stronger than the retention effect of the NLS domain. Reiterating the role of NES on the intracellular mobility of the ECFP, a restricted spread of blue colour was noticed when the NES was mutated to have weaker signalling (*NES-2*), wherein both blue and red fluorescence was found confined to the nuclear region alone. *NES-2* mutants had their leucine and isoleucine mutated to alanine, thereby losing the signalling property. This implied that altering the leucine level can knock out their functional potential since NES domains are generally leucine-rich [38]. This hypothesis was further supported by the *NES-1* mutation, which showed a stronger blue fluorescence in the cytoplasm in most of the systems except for *Ppmar2-NES-1* fused on the C-terminal. In *NES-1* mutation, glutamine and asparagine were mutated to leucine, increasing the leucine richness. Nevertheless, when there was no change in the leucine richness as in *NES-3* mutants, uniform blue fluorescence could be observed from both the nucleus and the cytoplasm. Among the *NES-3* mutants, in *Ppmar1-NES-3,* leucine was mutated to alanine and isoleucine, while proline was changed to glutamic acid*;* whereas in *Ppmar2-NES-3* serine was mutated to leucine. This could possibly render the *NES-3* mutants to intermediate signalling domains thereby bringing a balance in the protein distribution across the cell matrix. These observations from the cellular localization of the fluorescent proteins fused to NES of the TPases are suggestive of a parallel active role of the transposases in moso bamboo transposons *Ppmar1* and *Ppmar2*. Furthermore, there was no difference in export activity with respect to the site in which NES were fused to ECFP, either on C-terminal or N-terminal. Furthermore, the intensity of blue fluorescence observed for all of the mutants corresponded well with the signal values of the mutants predicted by the software, NetNES 1.1. as strong, weak, or intermediate.

### Excision of the transposons in yeast

In the excision frequency assay, yeast colonies can develop in Ade minimal medium only if the *Ade2* gene is expressed. Since *Ade2* is silenced in the vector by placing a non-autonomous MLE within the gene, excision of the transposon is essential for the *Ade2* expression. Therefore, the development of revertant yeast colonies can be directly associated with the rate of excision of the non-autonomous transposon from the *Ade2* gene. Since the excision is dependent on the TPase activity, the colony development can be further related to the Tpase efficiency which in turn is associated with the NES domain which is mutated. Although we have not directly observed transposition of moso bamboo MLEs in the present study, because we have not observed the insertion, from the transposition frequency assays for both *Ppmar1* and *Ppmar2*, we could gather the efficiencies of *NES* domains in both wild type and mutants in excising the transposon from the yeast genome. Since excision is an integral part of the transposition activity of the MLEs, the excision assay would, therefore, provide us with indirect evidence of transposition.

Among the NES mutants, we could find that *NES-1* mutants had produced very few revertant colonies than other systems, indicating that *NES-1* domains had relatively less amount of TPases in the nucleus to incite excision process. This also implied that TPases produced in the co-transformed yeast cells could have been exported out of the nucleus by the increased signalling activity of the *NES-1* domain. Therefore, *NES-1* domains were more than two times efficient than the wild type in preventing transposon excision. On the contrary, both *NES-2* and *NES-3* mutants had poor nuclear export signalling, due to which there was significantly high TPase activity in the nucleus and increased in transposon excision. This suggested that *NES-2* and *NES-3* mutants could have facilitated a relatively long residence time for the TPases in the nucleus. In the *Ppmar1-NES-3* mutant (L477A-P478E-L479I), the excision frequency was higher than *Ppmar1-NES-1* and *Ppmar1-NES-2*. However, among the *Ppmar2-NES*, excision frequency of *NES-3* mutant was lower than *NES-2* mutant.

The possible role of NESs of moso bamboo MLEs, *Ppmar1* and *Ppmar2* are to reduce their transposition activity. This can easily be illustrated from the fact that TPases are translated and produced in the cytoplasm and exported into the nucleus to bind to the target elements within the genome. However, with the weakening of the NES motif by mutation, the export machinery of TPases to the cytoplasm fails, and result in the nuclear accumulation of TPases, thereby triggering transposition [24]. Since the TPases are essential for the MLE transposition, their residence time in the nucleus could be an important factor determining the transposition frequency. A relatively long residence time would offer an increased chance of a TPase to bind to TIRs and excise the transposons [24]. Therefore, NES could be playing a significant role in controlling the residence time of TPase. In the rice MLE, *Ping* and *Pong*, which also possesses TPases harbouring NES domain [24], mutation of the NES domain was reported to have increased excision and transposition activity in rice and *Arabidopsis*. The mutant of *Ping* (*mPing*) had an increased activity by about six times when the *Ping* TPase-NES was weakened by mutation (L384/386A), while the *Pong* mutant had an increased excision and transposition activity by about 16 times following an NES mutation (L418/420A) [24]. In the same way, the NES mutant of Ozma TPase had resulted in an increased transposition activity of at least 4160 times for the *Eif* and *Goblin* MITEs in yeast [19].

By using non-autonomous mutants of *Ppmar1* and *Ppmar2*, we have decisively demonstrated that NES domains could regulate the transposon excision activity by regulating the nuclear export of TPase. The TEFs of all the NES mutants showed precise agreement with the NES export activity as observed in the fluorescence screening system. The activity of the wild type NES suggested that keeping the TPases less resident in the nucleus can prevent any undesired transposition activity, thereby minimizing any unwarranted genetic changes in the system so that the genome integrity could not be compromised. Since there is no NLS domain in the moso bamboo MLEs, we conclude that NES prominently regulates the transposition activity of these elements. Our results further suggest that TPases harbouring leucine-rich NESs domains are more efficient in maintaining genome integrity than those harbouring leucine-poor NES. Furthermore, to validate the hyperactivity of mutated NESs, they needed to be tested in the bamboo genome. This could help in developing active NES domains for tools of genetic manipulations and bamboo breeding.

## Conclusion

In conclusion, NES is an active domain in the *Ppmar1* and *Ppmar2* TPases of moso bamboo and the mechanism of NES is highly specific to the TPases. The present study has evidenced a significant function of the NES domain in the nuclear export of transposase, which could be important in maintaining genome integrity. Although NES domains are common in various genetic systems and mediate crosstalk between the cytoplasm and the nucleus either by interacting with signalling molecules or by their own movement [39, 40], there is no evidence any other additional functions to NES of moso bamboo MLEs other than controlling transposition. From the evidence, we speculate that NES could act as a regulatory switch to control the export of TPase and thereby control the transposon activation. We have also demonstrated that the NES domain itself can be easily be mutated to bring in changes in the nuclear export signalling. Therefore, we further speculate that the NES domain might have a function of maintaining the genome integrity under favourable conditions. On the contrary, the onset of unfavourable conditions could alter its function to create more mutations to increase cellular plasticity. Notwithstanding, these speculations of broader biological functions of NES needs further investigations, as conclusive evidence are still missing. TEs are abundant in the moso bamboo, and they play a major role in the genome evolution. Since transposon activity has evolutionary relevance, it would be interesting to understand the role of NES in genome evolution, spontaneous mutations and ultimately deciding the fitness and plasticity of the genome under selection pressure.

## Additional files

Additional file 1: The nucleotide sequences of *Ppmar1* and *Ppmar2* transposases and their amino acid sequences. (DOCX 18 kb)

Additional file 2: The sequences of primers used in the study to amplify the enhanced cyan fluorescent protein (ECFP) with a nuclear localization signal (NLS) (ECEP-NLS) and to mutate the NES sequences of *Ppmar1* and *Ppmar2* transposases. (DOCX 17 kb)

Additional file 3: The nucleotide sequences of *Ppmar1NA* and *Ppmar2NA* transposons (non-autonomous). (DOCX 15 kb)

Additional file 4: The sequences of primers used in the study for amplification of *Ppmar1NA*, *Ppmar2* and *Ppmar2NA* transposons. (DOCX 16 kb)

Additional file 5: The fluorescence intensity of both blue and red colour of ECFP and EYFP channels, respectively. (DOCX 18 kb)

## Data Availability

The data generated during this study and datasets supporting the conclusions are included in this article and its Additional file.

## Abbreviations

ECFP: Enhanced cyan fluorescent protein
EYFP: Enhanced yellow fluorescent protein
MLEs: Mariner-like elements
NES: Nuclear export signal
NLS: Nuclear translocation signal
ORF: Open reading frame
TEFs: Transposon excision frequencies
TEs: Transposable elements
TIRs: Terminal inverted repeats
TLEs: Tc1-like elements
TSDs: Target site duplicates
UTR: Untranslated region

## References

[1] Feschotte C, Jiang N, Wessler SR (2002). Plant transposable elements: where genetics meets genomics. Nat Rev Genet.

[2] Feschotte C, Pritham EJ (2007). DNA transposons and the evolution of eukaryotic genomes. Annu Rev Genet.

[3] Evgen’ev M (2007). Mobile elements and genome evolution. J Mol Biol.

[4] Emmons SW, Yesner L, Ruan K-s, Katzenberg D (1983). Evidence for a transposon in *Caenorhabditis elegans*. Cell..

[5] Jacobson JW, Medhora MM, Hartl DL (1986). Molecular structure of a somatically unstable transposable element in Drosophila. Proc Natl Acad Sci U S A.

[6] Hartl DL (2001). Discovery of the transposable element mariner. Genetics..

[7] Shao H, Tu Z (2001). Expanding the diversity of the IS630-*Tc1-*mariner superfamily: discovery of a unique DD37E transposon and reclassification of the DD37D and DD39D transposons. Genetics..

[8] Ammar I, Izsvak Z, Ivics Z (2012). The sleeping beauty transposon toolbox. Methods Mol Biol.

[9] Robert VJ (2012). Engineering the *Caenorhabditis elegans* genome by *Mos1*-induced transgene-instructed gene conversion. Methods Mol Biol.

[10] Tellier M, Bouuaert CC, Chalmers R (2015). Mariner and the ITm superfamily of transposons. Microbiol Spectr.

[11] Brezinsky L, Wang GV, Humphreys T, Hunt J (1990). The transposable element *Uhu* from Hawaiian Drosophila--member of the widely dispersed class of *Tc1-like* transposons. Nucleic Acids Res.

[12] Feschotte C, Wessler SR (2002). Mariner-like transposases are widespread and diverse in flowering plants. Proc Natl Acad Sci U S A.

[13] Tudor M, Lobocka M, Goodell M, Pettitt J, O'Hare K (1992). The *pogo* transposable element family of *Drosophila melanogaster*. Mol Gen Genet MGG.

[14] Lampe DJ, Grant TE, Robertson HM (1998). Factors affecting transposition of the *Himar1* mariner transposon in vitro. Genetics..

[15] Germon S, Bouchet N, Casteret S, Carpentier G, Adet J, Bigot Y (2009). *Mariner Mos1* transposase optimization by rational mutagenesis. Genetica..

[16] Lampe DJ, Akerley BJ, Rubin EJ, Mekalanos JJ, Robertson HM (1999). Hyperactive transposase mutants of the *Himar1* mariner transposon. Proc Natl Acad Sci U S A.

[17] Robertson H (1993). The mariner-like transposable element is widespread in insects. Nature..

[18] Ivics Z, Izsvak Z, Minter A, Hackett PB (1996). Identification of functional domains and evolution of *Tc1-like* transposable elements. Proc Natl Acad Sci U S A.

[19] Fattash I, Lee C-N, Mo K, Yang G (2015). Efficient transposition of the youngest miniature inverted repeat transposable element family of yellow fever mosquito in yeast. FEBS J.

[20] la Cour T, Kiemer L, Molgaard A, Gupta R, Skriver K, Brunak S (2004). Analysis and prediction of leucine-rich nuclear export signals. Protein Eng Des Sel.

[21] Xu D, Farmer A, Collett G, Grishin NV, Chook YM (2012). Sequence and structural analyses of nuclear export signals in the NESdb database. Mol Biol Cell.

[22] Demattei M-V, Hedhili S, Sinzelle L, Bressac C, Casteret S, Moiré N (2011). Nuclear importation of *mariner* transposases among eukaryotes: motif requirements and homo-protein interactions. PLoS One.

[23] Lohe AR, De Aguiar D, Hartl DL (1997). Mutations in the *mariner* transposase: The D,D(35)E consensus sequence is nonfunctional. Proc Natl Acad Sci U S A.

[24] Hancock CN, Zhang F, Wessler SR (2010). Transposition of the *Tourist-MITE* mPing in yeast: an assay that retains key features of catalysis by the class 2 PIF/Harbinger superfamily. Mob DNA.

[25] Zhou M-B, Lu J-J, Zhong H, Tang K-X, Tang D-Q (2010). Distribution and polymorphism of mariner-like elements in the Bambusoideae subfamily. Plant Syst Evol.

[26] Zhou M-B, Zhong H, Hu J-L, Tang D-Q (2015). *Ppmar1* and *Ppmar2*: the first two complete and intact full-length mariner-like elements isolated in *Phyllostachys edulis*. Acta Botanica Gallica.

[27] Zhou M, Hu H, Liu Z, Tang D (2016). Two active bamboo mariner-like transposable elements (*Ppmar1* and *Ppmar2)* identified as the transposon-based genetic tools for mutagenesis. Mol Breed.

[28] Zhou MB, Hu H, Miskey C, Lazarow K, Ivics Z, Kunze R (2017). Transposition of the bamboo mariner-like element *Ppmar1* in yeast. Mol Phylogenet Evol.

[29] Ramakrishnan M, Zhou M, Pan C, Hanninen H, Yrjala K, Vinod KK, et al. Affinities of terminal inverted repeats to DNA binding domain of transposase affect the transposition activity of bamboo *Ppmar2* mariner-like element. Int J Mol Sci. 2019;20(15):3692. 10.3390/ijms20153692 PMC6696609 31357686

[30] Fu SC, Huang HC, Horton P, Juan HF (2013). ValidNESs: a database of validated leucine-rich nuclear export signals. Nucleic Acids Res.

[31] Bryksin AV, Matsumura I (2010). Overlap extension PCR cloning: a simple and reliable way to create recombinant plasmids. Biotechniques..

[32] Anderson KI, Sanderson J, Gerwig S, Peychl J (2006). A new configuration of the Zeiss LSM 510 for simultaneous optical separation of green and red fluorescent protein pairs. Cytometry A.

[33] Schneider CA, Rasband WS, Eliceiri KW (2012). NIH image to ImageJ: 25 years of image analysis. Nat Methods.

[34] Michel K, Atkinson P (2003). Nuclear localization of the Hermes transposase depends on basic amino acid residues at the N-terminus of the protein. J Cell Biochem.

[35] Bleykasten-Grosshans C, Neuvéglise C (2011). Transposable elements in yeasts. C R Biol.

[36] Yang G, Weil CF, Wessler SR (2006). A rice *Tc1*/mariner-like element transposes in yeast. Plant Cell.

[37] Damelin M, Silver PA (2000). Mapping interactions between nuclear transport factors in living cells reveals pathways through the nuclear pore complex. Mol Cell.

[38] Mowen K, David M (2000). Regulation of STAT1 nuclear export by Jak1. Mol Cell Biol.

[39] McBride KM, McDonald C, Reich NC (2000). Nuclear export signal located within the DNA-binding domain of the STAT1transcription factor. EMBO J.

[40] García-Yagüe ÁJ, Rada P, Rojo AI, Lastres-Becker I, Cuadrado A (2013). Nuclear import and export signals control the subcellular localization of Nurr1 protein in response to oxidative stress. J Biol Chem.

