# Supplementary material for: Nuclear export signal (NES) of transposases affects the transposition activity of mariner-like elements Ppmar1 and Ppmar2 of moso bamboo
Source: Mob DNA. 2019 Aug 19;10:35. doi: 10.1186/s13100-019-0179-y (PMC6699137; doi:10.1186/s13100-019-0179-y)
Supplement: Supplementary file 1 — The nucleotide sequences of Ppmar1 and Ppmar2 transposases and their amino acid sequences. (DOCX 18 kb) [file 13100_2019_179_MOESM1_ESM.docx]

**Additional file 1.** The nucleotide sequences of *Ppmar1* and *Ppmar2* transposases and their amino acid sequences.

| **Name of the transposase** | **Nucleotide sequence of transposases (5’--3’)** |
| --- | --- |
| *Ppmar1* | TACTCCCTCCATACCCGAAATTCCTGACGTTTAGGACATGATTGTGGTAACCAAGGAGTGATTAATTAGGGGTTAGTTTTCCATCTTTGCCCCTAATAAATATGGTTACGGGTGCTCTTTGTACGAGAAAGTAAACCAGCTCGACTGGCTAGCGCGCGGAGGCCTCAGTCCTGTGGTGCGCGTTCGATACCTCGCGGACGCAGGTTTTTTTCTTGTTGCTGTTTATTCATTTTTGCATGGCACTGTTTAGGCAACGCACGTCGCGCGCGCTTAGCCGCTGCGGGCGTTAGTTTTCGAGTGGATTTGGGCCTGGCGCACGGAGGAGGTTGCATGGCTGCCCGAAAATTTCGTTGCATGCACTGGATTTTCAAAATTTTGTCCTCGCGCTGTGGAGGCTCGTTTGAGGCCGCGTTTTTTTTCATCTGGCGCGCTGGAAGGCCGACGTTTGGAGTGCTCGTTGCTTGTTCTATTTAAACGCCTGGAACCTTCCTTGTTGTCTTCCTATGCCGGACTCCTGTACTATGGCTGACCCAATAGATTCTGGCTTCGATCTGAACGTTCGGTTAGAAGAAGATGATGACGGCAATCTTCCCTTTGATCTCAACGAGCCAATATTGGAAGATCACAACAATGGTAAGCAAAAACGTCAAATTAGTTTCTCAGTTTCTCGTTTCCTTTTTTCTTTACTGAGCTTGTCGTTTCCTTTTTCGATAGGAATTGATTTGAACTTGCCATTAGATGAGTTTGGTGCTGTCGACTTCGACTATGTACAAAACCTCGCTGGTAAGCATGGCTAGTATTATGAATTCGCTTGTTTTTTTATTTCCTTTTGCTGGAACATGCCGTGAATAATAGTATTATGAACTCGCTTGTTTTTTATTTCCTTTTACTAGAACATGTGCTTGTTTTATTCCTATAGCTAGATCATGACGTCAATACTTTTTACGATGAATATGCTCGTTACAGTATAGCTAGAACATGCCGTGACTACATAGTAGTATGAATATGCTTGTTTTATTTCTATAACTATAACATGCCGTGAGTATATTTAGATCATGCCGTGAGTACTAAGTACTATTAAAATGCTTGTTTTTTATTTCCTTTTGCTAGAACAAGATGTTGAGGCTCCCGTTCAAGTACACCCTCCGAAGCATGACTATCCTGAACATGTTAGAAAACTAGTGTACCAAGCATTGTTGATGAGAAGCAAGAATGGGAAACTAGGCAATCATGATACAACAATTGTTTCCAGTCAATTTGGAGTAAAGATTCGATCAGTTCAGCGCATATGGAAGCAAGGTAAAAACCAACTTGCTCAAAACATTCCGGTCGTGGTTGCTAATCTAAAGAAAGGTAGAAGTGGCCGTAAAGCAACCCCTCTTGATTTGGAACAATTGCGCAACATTCCTCTCAAGCAAAGAATGACCATAGAAGATGTGTCTAGTAGACTTGGTATTAGCAAATCTAGGATACAAAGGTATTTGAAAAAGGGTTTGCTTAGGCGCCACTCTAGTAGCATAAAACCTTACCTCACCGATGCTAACAAGAAGACTAGGTTGAAGTGGTGCATTGACATGATTGAGCAAGGTTTGGTTGATGATCCAAAGTTCAGGGATTTGTTTGACTTTGTGTTTATTGATGAGAAGTGGTTCTACCTCTCTCAAAAATCCGAGAGATACTACTTGCTACCCGACGAAGATGAACCACATCGCACTTGCAAGAACAAGAATTACATCCCTAGGATCATGTTTTTGTGTGTTTGTGCTCGGCCAAGATTTAGAAATGGAGAATGTGTGTTTGATGGCAAAATAGGTTGTTTTCCACTAGTCACTTTTGAACAAGCTATTAGAGGAAGCCAAAACCGTCTTCGTGGAGAACAAGTAATCAAGCCAATTCAATCAATCAATAGGGAAGTGATAAGAGATTTCATGATAAATAGAGTGTTGCCTGCAATTAGAGCAAAGTGGCCAAGAGAAGATGTACACAAGCCAATTTTCATACAACAAGATAATGCTCCATCTCATTTAAAGGTGGATGATCCTCAGTTTTGTGAGGTTGCTAAGCAAGATGGGTTTGACATTAGGCTCATATGTCAACCACCCAATTCTCCAGATTTTAACATTCTAGATTTGGGTTTTTTTCGAGCTATTCAAGCAATTCAATACAAGAAAGATGCTAAGACATTGAAAGATCTAATTCCAGCAGTCCAACAGGTAAATGATCATCCATTACAGTGTTTAAATTGATCTTGAACAAATAATATAATCACTGATCTTGAACATGTTTTGTAGGCATTTTTGGAGTACTCTCCATGGAAAGCAAATAGGATATTTGTGACACTACAAACTGTTTTGAAGGAAGCAATGAAGATAAAAGGTTGCAACAAAATCAAAATTCCTCACATCCAGAAACAAAGACTTGAGAGAGAAGATAGGCTGCCATTGCAAATCCCTTGTGAAGCTTCCTTGCTAGCCGAAGCACTTGCAAGCCTTCCTGCGGCTAATTAGAAGATGCAAGCATGTTACTCTTTTGCAGCAGCAAGCATGTAAGAAGACGCGAGCATGTTAGTAGCAAACTATGAACAAACTAGTTTATGCATGTAGTAGTATGTTAGCTTGTGCACCTTAGTCATCTCGTCCCAACCGCTTGATAACATGCTCAGGAAGAAGTATTGTGTCACCATCCATTTCAAGTTTCTCCACATCAGGAATGTAGACCTCACAATCAAACTTTTCCATGTCATCGAGCCACTTCGCTGTCATGTCGTAGTCTTCATGTAAAAGGCCACAACGGGCACACATGCGAGCTTCGCGGCGAGCTTGGTAGCAGGCTTCTCCGAAGACGCCGCCGGCGTGGAACGTAACACAGCGAGGACACAGAGACTCGACGGAGTCGGGATCGACGGTGTCGGGCACCATCTCGAGGGAGTCTGCAACCATGTCGACGGAGTCCGGCAGCTCCTCGACGGAGTCCGGCACCATGTCGACGGTGTCCGGCAGCTCCTCGACGGAGTCTGGCACCTCCTGCGGCGCCATGTCCACGGTGTCCAGCGACGCTATGGAGCCCGACGAGATGTCCTGCACGGCGACGTCCAGCGCCGCAACGGACTCCGTCGTTTCCATCTGATCCGACGAGGCATCGACGTCCTGCGACGAGCGTGGCGGCGAGAGCACGGCGAGCGGGCAGGCGAGCGGGCAGGCGAGCGAGCCATTCGCGCGAGCGATGAATGCGAGCTGCTGTACCAGGCGCACACACGCGCAATCAATGCGGGCGAGTAACGATGCGAGCATGCGCGGCGGAAGCGCAACAGACGGGCAGCAGCGCATGGCCAGGGGCAAACGCGTGAAAAGAAGACCACGCGAGGCCACAACGTCAGCTTTTGCGCAAACGGGCACTTCGCCTAGAACGTCAGGAATTTCGGGTATGGAGGGAGTA |
| *Ppmar2* | TACTCCCTCCGTCCCAGTATAACGGGCGTATAACTTTTTTTACAGAGACCAAGGGACGGCGCGCGAGCGATGCATTCTTCTTCATCTACCCCTAATTAAACAGACGAGCGATGCATGCATGCAGTTTAATCACAAGCGATGCGTGCATGCAGATTTAAGAACGGCTTCTTTGCGCGCTCGCAGACCCACGTCTCACGCGCTCACACTCGTCGCCCACACTTGCTCGCACGCATGCATGACCAATCCTGTAGCAGTTAGAACGAGGATTATAGCGAAAATCACTTTGAACACAACATTTAGAAGATGGCGCCCAGAAATTGAGCTGGTGCGTATGGATTGAGAAGATTGCGCCCAGAAATCCAATTTCGTTTTCCTCCTATAAATAGGCATGAACACAACAGGTCTGTTGCCACTCTCTCCTCCTCTTCTCTCGTCCAGTTGCTTGCTCGAAATCAATGGTGAATTTGGACCTAAATCAACCTATCCATTGGGAAGAGATTGAGGACTATGATGGCCCTGTCATCGACCTTAATTTTGATCTTGTGTTTCATGATAGCAATGAAGGTATTACACAAATCCTTTATTCTGTTGTTTTGTTCCCCTAATGCTTACCGTTAGGATAGATCATGTATACTTGTTGTCTTGCTTGAAGAGGATGGCGGCCCGACCCATGGCGAAGAGGATGGTGGTGCACCTTCCCATGGAGAAGAGGACGGTGCCCCATCCCATGGCGAAGAGGATGGCACCCCTGCACCTAACGCCTATGAGACGAGTAAGACATGTCCTAGATTTTTGTATACTTGCTGCAGTAGGAGTGCGTATAACGAAGTAAATTGGGTTTGTTCGTATACAGTAGCTAGCTTGCTTGTTTGTTTGTATACATGTCCTAGAGTACATTAGCTTGCTTGCTGCACCAAAAAGTTTGAAACGGATTGGTTGTGCCTAGCAACAGAAAGAATTCTTGCACCTACCTTTGATTAATTTCTTTTTTTCCACACATTTGTAGATCAAGTTGGCAATGTTAGAAGGAGGAATCAATATACTGTGATCAACGCCGAGCCATATATAGCATGCTTCTAGGGCGAACTTCTGTCGGCATATTGAAGAAAGGAGCGACGAAGgCCGTTTCTGTTGAAACAGGCGTGCCTTTGAGAGTTGTGCAACGGATATGGCTAAACGGACAGTAGGGTGGTGGGGTAAACGCAGTCTCTAGCAAGAAGGCAAAGAATTGTGGCTGGAAACGAGTAGCCTTCGATCCGGAAGCCATCAAAGATGTGCCTTTGAGTAGCCGAACGACAATCCGGGATCTAGCAGGCGCTCTGAATATTTCAAAGAGCACATTGTTTAGGCAGATGAAAGAAGGGAAGTTTAGACGGCACACAAATGACATTAAGTTTACATTGACTGAAGATAACAAGAAAGCATGTGTTAAGTTTTGCCTCTCAATGCTAGAAAAATTAAGCATGCCGCAAGAACCAACTTTTGAGGGTATGTACAACATCGTGTACATAGACGAAAAGTGGTTCTATCGGATGAGGAAATTTCAAAACTACTACTTGGCGCCAGATGAGGACAAGCCAGAAAGAACCACAAAGAGTAAAAATTTCATAGAGAAGGTGATGTTGCTCGCAGAAATTGCGAGACCTAAATTTGATTGGGATGGAAATGTTACATTTTCTGGAAGGATAGGCATAATTCCTTTCACTTTCGTAGAGCTAGCAAAGCGAAGTAGTGCGAATAGGCCTGCTGGTACATTGGTGACCAAGGCAATGACATCGGTAACCAAGGAAACAAGCCGTGAGTACCTTGTAAATAAGGTATTGCCCGCGATCAAGCAAAAATGGCTAGCGGAGGAAGTTGGTACCCCCATATTCATCCAGCAGGATAATGCTAGGACGCATATTGCAATCAATGATGACGAGTTTTGTCGTGCGGCATCCGCAGATGGTTTTGACATAAGTTTGATGTGCCAGCCACCCAACTCTCCTGATCTCAATGTATTAGATCTTGGTTTTTTTGCGGCCATTCAATCCATGTTTCAAAAGTCGTCTCCAAGCAACATTGAAGACATTGTTGCCAAGGTAATCCAAGCTTTTGACGAGTATCCAGTTGATAGGAGTAACCGTATTTTCCTCACTCACCAATCATGCATGAGAGAAATTTTGCGTCAAAAAGGAGGGCAACACTATGCAATCCCACACTTGAAGAAGCAATCACTTGAGAGGAATGGTGTTCTTTCCATTAGATTACAATGTGACCTAGTAGTTGTGAATGAAGCAATTGTGTACATCAATTAGAATCTGTTATTGCAAATGTAATCAAACCTGTACTGTTTTTCCTTGCTTAAGCTAGATTTGTCAAACAAGTAAATTTGTTGTGTTCAAACCATAGTTAGATTTGTCAAACAAGTACGCCACATGTGTAGAAAATGAGTAGTACATCAAACGAGTAGCCAGAATTTTTTGTTACCATTCACACAGTTTATTCTTCATCGTTCTCGATAAAGGCGTCCTCCACAGTGCACTCGACATCTGCAAGGGACTCCGGGACATACGAGTTGCCGGCAAGAGAGTCTACATCCATGTAGCTTTGCTTATCGTCGCTGTCGTCAATCCCGGGTAGGAGTATATTGCAGTCGAACTCGTCGAGGCCGTAGATCCAAGCCGTGACCATGTAATCACAGTGGACGAGACCACAGCGAGAGCACGAACCCTTCAATACGCGCCTCTCATCAAGGAGCTTGTCACTCCTGGACGCGTCAGGGTCCTTCATGTTATCGTTTTCACGTGCGTCAATGTCGTCGGTGTCGTCGAGCCCGGCAAGGAGGGTGTCATCGGTGTCATGCACGCACTTCTGCTTGTGGTTGCCATCATCGAGATCGATGGGCACGGGCTTCTGCTTGTGGTCGCCGTCGTCGAGAATGCCTATGAGTGCGGGCTTCTGCTTGCGGTCGCCATCGTCGAGATCAGTGAGCGTGGGCTTCTGCTTGTGGTCGTCGTTGTCGAGATCGACGAGCGTGGGCTTCTGGTTGTCGTCTGCAAGGGACTTCAGGACATACGAGTTGCCAGTAAGAGAGTCTACATCCATGTAGCTTTGCTTATCGTCGCTGTCGTCAATCCCGGGTAGGAGTATATTGCAGTCAAACTTGTCGAGGCCGTAGATCCAAGCCATGACCATGTAATCGCAGTGGACGAGACCACAGCGAGAGCACGAACCCTTCAATACGCACCTCTCATCAAGGAGCTTGTCGCTGCTGGACGCGTCGGGGTCCTTCTTGTCATCGTTTTCACGTGCGTTAGTGTCGTCAGTGTCGTCGAGCCCGACAAGGAGGGTGTCATCGGCGTCGTGCACGCTCTTCTGCTTGTGGTTGCCGTCGTCGAGATCGATGGGCACGGGCTTCTGCTTGTGGTCGCCGTCGTCGAGATCGGCGAGCGCCGGCTTCTGCTTGTGGTCGCCGTCGTCGAGAATGCCTATGAGTGTGGGCTTCTGCTTGCGGTCGCCATCATCGAGATCAGTGAGCGCAGGCTTCTGCTTGTGGTCACCGTCATCGAGATGGCAAGCGCGGGCTTCTCCTTGTGGTCGCCCTCCTCACGACCACCCGGGGTCTCAGCGGCCATCACCGTGGAACTCACTCAATCGGGGTCTTCAATTGGGCCGATTAAAATGGATCAGTAACAGTGGCTCTAAGCGTCGCATGCATCACGCGTATTCCTCACACTTTTCTAAGTGGTCGCATGCATCACGCGTCAAGCAGTCATTACTGGCACTTCTCTGAGCAGTTGCATGCATCACGCGTTAAGCAGTACCACTTCTCTAAGCGTCACGCATGCATGCATGCATGCAGATTGCTGAGGGCAAAGATGGGAGAAAGCAGCGCAAAATGAAGGGACGCCTTGTATTGTGGGACAGCAGAAAAAAAGTTATATGCCCGTTATACTGGGACGGAGGGAGTA |
|  | **Amino acid sequences of transposase** |
| *Ppmar1* | MADPIDSGFDLNVRLEEDDDGNLPFDLNEPILEDHNNGIDLNLPLDEFGAVDFDYVQNLAEQDVEAPVQVHPPKHDYPEHVRKLVYQALLMRSKNGKLGNHDTTIVSSQFGVKIRSVQRIWKQGKNQLAQNIPVVVANLKKGRSGRKATPLDLEQLRNIPLKQRMTIEDVSSRLGISKSRIQRYLKKGLLRRHSSSIKPYLTDANKKTRLKWCIDMIEQGLVDDPKFRDLFDFVFIDEKWFYLSQKSERYYLLPDEDEPHRTCKNKNYIPRIMFLCVCARPRFRNGECVFDGKIGCFPLVTFEQAIRGSQNRLRGEQVIKPIQSINREVIRDFMINRVLPAIRAKWPREDVHKPIFIQQDNAPSHLKVDDPQFCEVAKQDGFDIRLICQPPNSPDFNILDLGFFRAIQAIQYKKDAKTLKDLIPAVQQAFLEYSPWKANRIFVTLQTVLKEAMKIKGCNKIKIPHIQKQRLEREDRLPLQIPCEASLLAEALASLPAAN |
| *Ppmar2* | MANLDLNQPIHWEEIEDYDGPVIDLNFDLVFHDSDEGDGGPTHGEEDGGAPSHGEEDGGPTHGEEDGGAPSHGEEDGAPSHGEEDGTPAPNAYETISTNKAKNCGWKRVAFDPEAIKDVPLSSRTTIRDLAGALNISKSTLFRQMKEGKFRRHTNDIKFTLTEDNKKACVKFCLSMLEKLSMPQEPTFEGMYNIVYIDEKWFYRMRKFQNYYLAPDEDKPERTTKSKNFIEKVMLLAEIARPKFDWDGNVTFSGKIGIIPFTFVELAKRSSANRPAGTLVTKAMTSVTKETSREYLVNKVLPAIKQKWLAEEVGTPIFIQQDNARTHIAINDDEFCRAASADGFDISLMCQPPNSPDLNVLDLGFFAAIQSMFQKSSPSNIEDIVAKVIQAFDEYPVDRSNRIFLTHQSCMREILRQKGGQHYAIPHLKKQSLERNGVLSIRLQCDLVVVNEAIVYIN |
