# Supplementary material for: Nuclear export signal (NES) of transposases affects the transposition activity of mariner-like elements Ppmar1 and Ppmar2 of moso bamboo
Source: Mob DNA. 2019 Aug 19;10:35. doi: 10.1186/s13100-019-0179-y (PMC6699137; doi:10.1186/s13100-019-0179-y)
Supplement: Supplementary file 2 — The sequences of primers used in the study to amplify the enhanced cyan fluorescent protein (ECFP) with a nuclear localization signal (NLS) (ECEP-NLS) and to mutate the NES sequences of Ppmar1 and Ppmar2 transposases. (DOCX 17 kb) [file 13100_2019_179_MOESM2_ESM.docx]

**Additional file 2.** The sequences of primers used in the study to amplify the enhanced cyan fluorescent protein (ECFP) with a nuclear localization signal (NLS) (ECEP-NLS) and to mutate the NES sequences of *Ppmar1* and *Ppmar2* transposases.

| **Primer** | **Sequences （5’--3’）to amplify ECEP-NLS** |
| --- | --- |
| ECFP-NLS-F | ATAAGAATGCGGCCGCCTATGGTGAGCAAGG (plus *Not Ⅰ* Restriction sites (GCGGCCGC)) |
| ECFP-NLS-R | CCGCTCGAGCTAGCAGCCGGATCCCTTGTA (plus XhoⅠ Restriction sites (CTCGAG)) |
| **Primer** | **Sequences （5’--3’）to mutate NES of *Ppmar1* transposase** |
| A477L-A479L-F | AGAAACAAAGACTTGAGAGAGAAGATAGGCTGCCATTGCAAATCCCTTGTGAAGCTTCCTTGCTAGC |
| A477L-A479L-R | GCTAGCAAGGAAGCTTCACAAGGGATTTGCAATGGCAGCCTATCTTCTCTCTCAAGTCTTTGTTTCT |
| Z-E472I-F | CTCACATCCAGAAACAAAGACTTATCAGAGAAGATAGGCTGCCATTG |
| Z-E472I-R | CAATGGCAGCCTATCTTCTCTGATAAGTCTTTGTTTCTGGATGTGAG |
| Z-Q469L-F | AAAATTCCTCACATCCAGAAACTGAGACTTGAGAGAGAAGATAGGC |
| Z-Q469L-R | GCCTATCTTCTCTCTCAAGTCTCAGTTTCTGGATGTGAGGAATTTT |
| Z-A477L-F | AGACTTGAGAGAGAAGATAGGCTGCCAGCTCAAATCCCTTGTGAA |
| Z-A477L-R | TTCACAAGGGATTTGAGCTGGCAGCCTATCTTCTCTCTCAAGTCT |
| Z-P478E-A479I-F | CAAAGACTTGAGAGAGAAGATAGGGCTGAAATTCAAATCCCTTGTGAAGCTTCC |
| Z-P478E-A479I-R | GGAAGCTTCACAAGGGATTTGAATTTCAGCCCTATCTTCTCTCTCAAGTCTTTG |
| **Primer** | **Sequences （5’--3’）to mutate NES of *Ppmar2* transposase** |
| X-I441A-F | TTGAGAGGAATGGTGTTCTTTCCGCTAGATTACAATGTGACCTAGTAG |
| X-I441A-R | CTACTAGGTCACATTGTAATCTAGCGGAAAGAACACCATTCCTCTCAA |
| X-S440L-F | CAATCACTTGAGAGGAATGGTGTTCTTCTGATTAGATTACAATGTGACCTAGTAGTT |
| X-S440L-R | AACTACTAGGTCACATTGTAATCTAATCAGAAGAACACCATTCCTCTCAAGTGATTG |
| X-C445L-F | CTTGAGAGGAATGGTGTTCTTTCCATTAGATTACAACTGGACCTAGTAGTTGTGAATG |
| X-C445L-R | CATTCACAACTACTAGGTCCAGTTGTAATCTAATGGAAAGAACACCATTCCTCTCAAG |
| X-N436L-F | ACTTGAAGAAGCAATCACTTGAGAGGCTGGGTGTTCTTTCCATTAGATTACAATG |
| X-N436L-R | CATTGTAATCTAATGGAAAGAACACCCAGCCTCTCAAGTGATTGCTTCTTCAAGT |
