# Supplementary material for: Nuclear export signal (NES) of transposases affects the transposition activity of mariner-like elements Ppmar1 and Ppmar2 of moso bamboo
Source: Mob DNA. 2019 Aug 19;10:35. doi: 10.1186/s13100-019-0179-y (PMC6699137; doi:10.1186/s13100-019-0179-y)
Supplement: Supplementary file 3 — The nucleotide sequences of Ppmar1NA and Ppmar2NA transposons (non-autonomous). (DOCX 15 kb) [file 13100_2019_179_MOESM3_ESM.docx]

**Additional file 3.** The nucleotide sequences of *Ppmar1NA* and *Ppmar2NA* transposon (non-autonomous).

| **Name of the transposon** | **Nucleotide sequence of transposon（5’--3’）** |
| --- | --- |
| *Ppmar1NA* | TACTCCCTCCATACCCGAAATTCCTGACGTTCTAGGCGAAGTGCCCGTTTGCGCAAAAGCTGACGTTGTGGCCTCGCGTGGTCTTCTTTTCACGCGTTTGCCCCTGGCCATGCGCTGCTGCCCGTCTGTTGCGCTTCCGCCGCGCATGCTCGCATCGTTACTCGCCCGCATTGATTGCGCGTGTGTGCGCCTGGTACAGCAGCTCGCATTCATCGCTCGCGCGAATGGCTCGCTCGCCTGCCCGCTCGCCTGCCCGCTCGCCGTGCTCTCGCCGCCACGCTCGTCGCAGGACGTCGATGCCTCGTCGGATCAGATGGAAACGACGGAGTCCGTTGCGGCGCTGGACGTCGCCGTGCAGGACATCTCGTCGGGCTCCATAGCGTCGCTGGACACCGTGGACATGGCGCCGCAGGAGGTGCCAGACTCCGTCGAGGAGCTGCCGGACCATGCAACCTCCTCCGTGCGCCAGGCCCAAATCCACTCGAAAACTAACGCCCGCAGCGGCTAAGCGCGCGCGACGTGCGTTGCCTAAACAGTGCCATGCAAAAATGAATAAACAGCAACAAGAAAAAAACCTGCGTCCGCGAGGTATCGAACGCGCACCACAGGACTGAGGCCTCCGCGCGCTAGCCAGTCGAGCTGGTTTACTTTCTCGTACAAAGAGCACCCGTAACCATATTTATTAGGGGCAAAGATGGAAAACTAACCCCTAATTAATCACTCCTTGGTTACCACAATCATGTCCTAAACGTCAGGAATTTCGGGTATGGAGGGAGTA |
| *Ppmar2NA* | TACTCCCTCCGTCCCAGTATAACGGGCGTATAAAAAAATTTCTGCTGTCCCACAATACAGGGCGTCCCTTCAATTTTGCACCGCTTTCTCCCATTTTGCCCTCAGCAATCTGCATGCATGCATGCATGCGTGACGCTTAGAGTTCTGCTTAACCGCGTGATGCATTCAACTGCTCTGAGAAGTGCCAGCGTGATGCATGCCACTGCTTAGAAAAGTGCGAGGATCCGGTGACACGACCATCATAGTCCTCAATCTCTTCCCAATGGATAGGTTGATTTAGGTCCAAATTCGCCATTGATTTCGAGCAAGCAACTGGACGAGAGAAGAGGAGAGAGTGGCAGCAGACCTGTTGTGTTCATGTCTATTTATAGGAGGAAAACGAATTTGAATTTCTGGGGGCAATCTTCTCAATCCATATGCACTAGCTCAATTTCTAGGCGCCATCTTCTAAATGCTGTGTTCAAATTTTCGCTATAATCCGCTATAGACTTCTAAATGCTGGGCGCCATCTTCTAAATGCATGCGTGCGAGCGAGTACGAGCCTACGAGCGCGCGAATCTGCATGCATCGCTCAAATCTGCATGCCATGCTTCTCTCGCGTGTGAATCTGCATGCCATGCTTCTCTCGCGCACGAATCTGCATGCTGTGCTTTAAATCTGCATGCCGTGCTTTAAATCTGCATGCATGCATGGTCATTGATTATAGCAAACTGCACGCATGCATCTCATCAATTTAATTAGGGGTAGATGAGGAAGAATCGCTAGCTCGCGCGCCCTCCTTGGTCTCTGAAATTTTTTTATACGCCCGTTATACTGGGACGGAGGGAGTA |
