# Supplementary material for: Nuclear export signal (NES) of transposases affects the transposition activity of mariner-like elements Ppmar1 and Ppmar2 of moso bamboo
Source: Mob DNA. 2019 Aug 19;10:35. doi: 10.1186/s13100-019-0179-y (PMC6699137; doi:10.1186/s13100-019-0179-y)
Supplement: Supplementary file 4 — The sequences of primers used in the study for amplification of Ppmar1NA, Ppmar2 and Ppmar2NA transposons. (DOCX 16 kb) [file 13100_2019_179_MOESM4_ESM.docx]

**Additional file 4.** The sequences of primers used in the study for amplification of *Ppmar1NA, Ppmar2* and *Ppmar2NA* transposons.

| **Primer** | **Sequences （5’--3’）** | **Function** |
| --- | --- | --- |
| Mini-3-1F | TACTCCCTCCATACCCGAAATTCCT | For amplification of the *Ppmar1NA*. |
| Mini-3-1R | TACTCCCTCCATACCCGAAATTCCT |  |
| Mini-s-1-20-1F | CCGCTCGAGTACTCCCTCCGTCCCAGTATAACGG | For amplification of the 5’ TIR and flanking sequences of *Ppmar2* |
| Mini-s-1-20-1R1 | TGATGGTCGTGTCACCGGATCCTCGCACTTTTCT |  |
| Mini-s-1-20-2F1 | GGTGACACGACCATCATAGTCCTCAATCTC | For amplification of the 3’ TIR and flanking sequences of *Ppmar2* |
| Mini-s-1-20-2R | CCGCTCGAGTACTCCCTCCGTCCCAGTATAACGG |  |
| Mini-s-1-20-1F | CCGCTCGAGTACTCCCTCCGTCCCAGTATAACGG | For amplification of *Ppmar2NA* |
| Mini-s-1-20-2R | CCGCTCGAGTACTCCCTCCGTCCCAGTATAACGG |  |
