# Supplementary material for: Nuclear export signal (NES) of transposases affects the transposition activity of mariner-like elements Ppmar1 and Ppmar2 of moso bamboo
Source: Mob DNA. 2019 Aug 19;10:35. doi: 10.1186/s13100-019-0179-y (PMC6699137; doi:10.1186/s13100-019-0179-y)
Supplement: Supplementary file 5 — The fluorescence intensity of both blue and red colour of ECFP and EYFP channels, respectively. (DOCX 18 kb) [file 13100_2019_179_MOESM5_ESM.docx]

**Additional file 5.** The fluorescence intensity of both blue and red colour of ECFP and EYFP channels, respectively.

| **Terminal position** | **Types of NES** | **Red fluorescence** | **Blue fluorescence** |
| --- | --- | --- | --- |
| NES-ECFP | *Ppmar1-NES-0* | 26.463 ± 3.536 | 61.818 ± 6.832 |
|  | *Ppmar1-NES-1* | 21.1 ± 3.336 | 56.024 ± 9.963 |
|  | *Ppmar1-NES-2* | 22.422 ± 5.726 | 25.616 ± 7.625 |
|  | *Ppmar1-NES-3* | 22.252 ± 2.345 | 43.526 ± 8.087 |
| ECFP-NES | *Ppmar1-NES-0* | 17.573 ± 4.282 | 44.898 ± 3.027 |
|  | *Ppmar1-NES-1* | 19.84 ± 5.101 | 66.011 ± 6.591 |
|  | *Ppmar1-NES-2* | 18.764 ± 2.321 | 36.042 ± 4.186 |
|  | *Ppmar1-NES-3* | 19.813 ± 4.661 | 38.435 ± 7.018 |
| NES-ECFP | *Ppmar2-NES-0* | 18.447 ± 4.713 | 39.259 ± 7.928 |
|  | *Ppmar2-NES-1* | 25.373 ± 5.156 | 49.357 ± 2.655 |
|  | *Ppmar2-NES-2* | 27.373 ± 4.565 | 50.207 ± 5.637 |
|  | *Ppmar2-NES-3* | 23.496 ± 1.461 | 33.889 ± 5.628 |
| ECFP-NES | *Ppmar2-NES-0* | 24.773 ± 3.291 | 38.761 ± 4.188 |
|  | *Ppmar2-NES-1* | 27.42 ± 3.636 | 60.594 ± 7.324 |
|  | *Ppmar2-NES-2* | 30.524 ± 5.57 | 47.024 ± 2.48 |
|  | *Ppmar2-NES-3* | 25.774 ± 4.262 | 37.816 ± 2.304 |

ECFP, enhanced cyan fluorescent protein; EYFP, enhanced yellow fluorescent protein; NES-ECFP, NES fused in the N-terminal of ECFP; ECFP-NES, NES fused in the C-terminal of ECFP.
